# The Abuse of the Forceps

**Published:** 1897-09-18

**Authors:** 


### The Abuse of the Forceps.

It would be difficult to over-estimate the gravity of the charge brought against the modern practice of midwifery by Professor Japp Sinclair in the address recently delivered by him at Montreal. What he says is, in effect, that recourse is far too often had to instrumental aid, that the forceps are applied very much more frequently than is either necessary or desirable, that they are used not altogether for the sake of the patient but rather with the object of saving time, and that this "meddlesome and mischievous" midwifery causes much injury and leads to serious after consequences.

Gynæcology, he says, flourishes, and has become largely surgical, but this is the direct result of surgical interference in midwifery practice. The accoucheurs are the providers of the material for the gynæcologists. Much attention has been directed to the subject of avoidable childbed mortality, but the very serious question of childbed morbidity has also to be considered, and this he maintains is largely owing to the prevalence of surgical methods in the practice of midwifery.

Now, we would recall attention to the fact that Dr. Cullingworth, in his address to the Obstetrical Society last March on the Undiminished Mortality from Puerperal Fever in England and Wales, pointed to Lancashire and Cheshire as holding an unenviable pre-eminence in the black list, and it becomes a matter of very great interest and importance to observe, as a possible contributory cause, the great frequency with which, according to Professor Sinclair, forceps are applied in that part of the country, especially in view of the injuries which he considers so often follow their use. "In Manchester and the manufacturing towns of Lancashire the proportion of cases in which the forceps are applied, with or without indications, amounts to 25 or 30 per cent., and even more. One of my friends who has a large general practice within the area covered by our Maternity Hospital has been good enough to give me a statement of his midwifery practice for the last 10 years, and the proportion comes as nearly as possible to 25 per cent. From 1885 to 1889 (5 years) he attended 839 cases, and applied the forceps in 142; that is, in 17 per cent. From 1890 to 1896 (7 years) he attended 900 cases, and used the forceps in 246; that is, a percentage of 27·3. His rate of forceps delivery is greatest in 1896, when he used the instrument 50 times in 150 cases. Another friend, whose practice lies within the same area, tells me that his proportion is at least 30 per cent. The highest figure mentioned to me has been 75 per cent. A busy practitioner, whose field of operation lies in one of the largest manufacturing towns in Lancashire, told me, in answer to the question I so frequently put, "What is the percentage of forceps cases in your practice?" that his was "at least 75 per cent." "But," I replied, "you must be joking." "Not at all," he said, "between high and low applications of the forceps, at least 75 per cent." Now it may be said that this is but one more sad proof of the prevalence of pelvic deformities in a community of factory operatives. But when we come to inquire what are the conditions which are there considered to demand operative interference we find nothing to bear out such a supposition; it would appear to be a mere matter of impatience. "I have been frequently told by practitioners in similar communities, that in the case of a multipara they allow half an hour for the second stage of labour, and if the case does not show signs of immediate spontaneous completion they apply the forceps." And again, "Among the gynæcological cases at the Manchester Southern Hospital, it is by no means a rare thing to find a young woman suffering from dislocation of the uterus and laceration of the cervix and of the perineum, whose first labour was terminated by forceps within four to six hours of the onset of regular pains."

This is the charge brought against the midwifery of the north of England by the Professor of Obstetrics at Owen's College, but it is not all. In our view of the matter, a still more serious charge is brought against the medical practitioners of that part of the country, at least by implication, in the following sentence: "Another point, which I mention with some diffidence, because I have only my own figures to offer by way of illustration, is the remarkable difference in the proportion of forceps deliveries among the poor as compared with those in a better position in life. I have for a long time made cautious inquiries with regard to the history of the confinements in taking notes of my private gynæcological cases, and my conclusion is that the hospital patients are delivered with forceps more than ten times as often as the class of women who consult the gynæcologist privately, and may therefore be assumed to be in a position to pay higher fees to the accoucheur. If this result should be found, on extended inquiry, to coincide with the experience of others in a similar position, it is a not unimportant fact in guiding our judgment to a conclusion as to how far we may have drifted astray from right and reasonable midwifery practice at the present time, and as to one cause at least of the aberration."

There can, we think, be no doubt about the meaning of this sentence, viz., that in cheap midwifery practice forceps are used merely to save time. This comes out all the more strongly when we compare the figures given as to the frequency with which they are employed in working-class private practice with those as to the same point derived from the Manchester Maternity, where it seems that, including both in and out patients, the number of forceps cases was under 2·6 per cent. of the whole. Clearly, the practitioners of Manchester cannot remain indifferent to such a charge as this. They must either refute it or take steps to bring the practice of midwifery in their district more into harmony with general teaching.

